# AI-Generated Patient-Friendly MRI Fistula Summaries: A Pilot Randomised Study

**DOI:** 10.3390/jimaging11090302

**Published:** 2025-09-04

**Authors:** Easan Anand, Itai Ghersin, Gita Lingam, Theo Pelly, Daniel Singer, Chris Tomlinson, Robin E. J. Munro, Rachel Capstick, Anna Antoniou, Ailsa L. Hart, Phil Tozer, Kapil Sahnan, Phillip Lung

**Affiliations:** 1 Robin Phillips’ Fistula Research Unit, St Mark’s The National Bowel Hospital, London NW10 7NS, UK; itai.ghersin@nhs.net (I.G.); gita.lingam@nhs.net (G.L.); h.pelly@nhs.net (T.P.); ailsa.hart@nhs.net (A.L.H.); philtozer@nhs.net (P.T.); kapil.sahnan@nhs.net (K.S.); philliplung@nhs.net (P.L.); 2 Department of Surgery & Cancer, Imperial College London, London SW7 2AZ, UK; 3 Tenrec Analytics, St Albans AL1 4TJ, UK; daniel.singer@tenrecanalytics.com; 4 Institute of Health Informatics, University College London, London NW1 2DA, UK; christopher.tomlinson@ucl.ac.uk; 5 Patient Advocate, St Mark’s The National Bowel Hospital, London NW10 7NS, UK; web@robinmunro.com (R.E.J.M.); rachelcapstick@hotmail.com (R.C.); anna.antoniou@gmail.com (A.A.)

**Keywords:** artificial intelligence, Crohn’s disease, large language models, magnetic resonance imaging, patient communication, perianal fistula

## Abstract

Perianal fistulising Crohn’s disease (pfCD) affects 1 in 5 Crohn’s patients and requires frequent MRI monitoring. Standard radiology reports are written for clinicians using technical language often inaccessible to patients, which can cause anxiety and hinder engagement. This study evaluates the feasibility and safety of AI-generated patient-friendly MRI fistula summaries to improve patient understanding and shared decision-making. MRI fistula reports spanning healed to complex disease were identified and used to generate AI patient-friendly summaries via ChatGPT-4. Six de-identified MRI reports and corresponding AI summaries were assessed by clinicians for hallucinations and readability (Flesch-Kincaid score). Sixteen patients with perianal fistulas were randomized to review either AI summaries or original reports and rated them on readability, comprehensibility, utility, quality, follow-up questions, and trustworthiness using Likert scales. Patients rated AI summaries significantly higher in readability (median 5 vs. 2, *p =* 0.011), comprehensibility (5 vs. 2, *p =* 0.007), utility (5 vs. 3, *p =* 0.014), and overall quality (4.5 vs. 4, *p =* 0.013), with fewer follow-up questions (3 vs. 4, *p =* 0.018). Clinicians found AI summaries more readable (mean Flesch-Kincaid 54.6 vs. 32.2, *p =* 0.005) and free of hallucinations. No clinically significant inaccuracies were identified. AI-generated patient-friendly MRI summaries have potential to enhance patient communication and clinical workflow in pfCD. Larger studies are needed to validate clinical utility, hallucination rates, and acceptability.

## 1. Introduction

**Figure 1:**
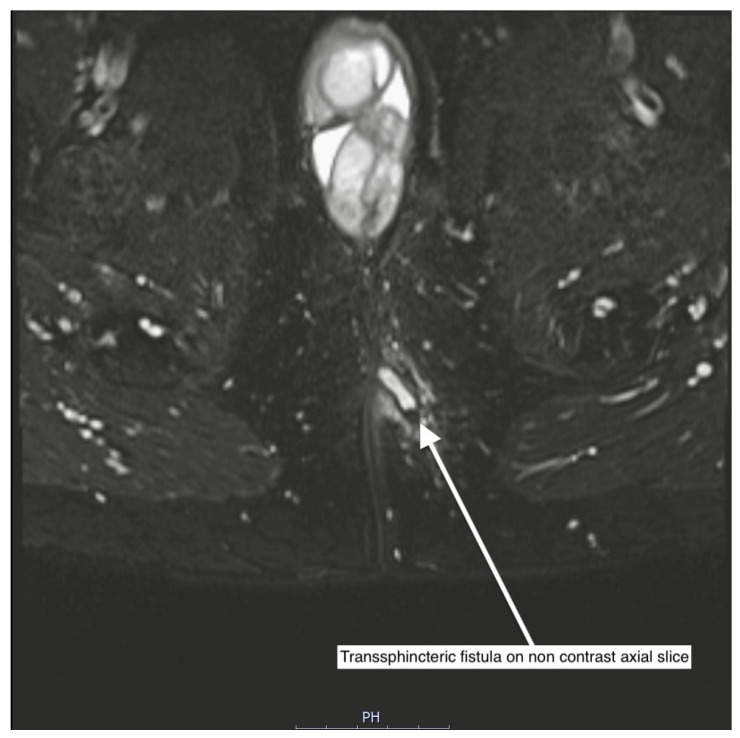
An example non-contrast T2 fat-saturated axial slice from an MRI scan showing a transsphincteric fistula in perianal fistulising Crohn’s disease.

Perianal fistulising Crohn’s disease (pfCD) affects approximately 1 in 5 Crohn’s disease patients and requires repeated MRI imaging for monitoring [1,2]. MRI reports are traditionally written for clinicians using technical language, which patients find difficult to understand, especially with direct access to reports via digital platforms like the NHS application [3,4]. Figure 1 illustrates an axial (transverse) slice from a T2 fat-saturated MRI sequence in a patient with complex pfCD.

AI, particularly Large Language Models (LLMs), offers promise in generating patient-friendly summaries to improve comprehension and engagement [5,6,7]. This study evaluates the feasibility and safety of AI-generated patient-friendly MRI summaries for pfCD patients, aiming to improve communication and shared decision-making.

## 2. Materials and Methods

A Patient and Public Involvement (PPI) day and global survey identified the need for patient-friendly MRI summaries [3]. A PACS database search identified six de-identified MRI fistula reports of varying complexity. Cases were selected by two independent clinical researchers and approved by the senior authors to represent a broad spectrum of disease states, ranging from completely healed fistulae to complex fistulae with multiple tracts and complicating features. This approach was taken to minimise case selection bias and ensure representative variation in disease complexity. Prompt engineering was conducted with three patient representatives and one AI industry expert to ensure concise, patient-centred prompts. AI-generated summaries were created using ChatGPT-4 with a standardized prompt: “In each response, please write a short patient-friendly summary appropriate for a reading age of 12 (aiming for a Flesch-Kincaid score of 60+) where the findings of the report are succinctly summarised, and there is a meaningful actionable recommendation at the end.” Two clinicians independently assessed the AI summaries and original reports for hallucinations, completeness, and readability (Flesch-Kincaid score). Hallucinations were defined as content not supported by or contradictory to the source report, and assessments were performed independently by two reviewers with consensus reached after discussion; any discrepancies were resolved with input from senior authors.

Sixteen patients with perianal fistulas were recruited and randomized to review either AI summaries or original reports.

Below is an example of an original radiology report and of an AI-generated patient-friendly report of the same case. A further example of a complex fistula report and AI-generated summary is included in Appendix A.

Original report:
  “Comparison is made with previous MRI pelvis
  Distortion, poor definition and altered signal of the internal sphincter noted posteriorly from 5 o’clock to 7 o’clock positions in the lower canal level, which is likely to represent a chronic sequelae of past inflammation—appearances are unchanged compared to the previous examination. Otherwise normal appearance of the anal sphincters and anal canal with no evidence of active perianal sepsis or fistula.
  Rectal appearances are consistent with known proctitis which is also unchanged. No ischio-anal or supralevator sepsis. No demonstrable abnormality of the urinary bladder, urethra, prostate or seminal vesicles. Other pelvic organs included in the scan volume show no obvious abnormality. No pelvic or inguinal lymphadenopathy. No pelvic free fluid. Bilateral small uncomplicated fat containing inguinal hernias are noted (inguinal region not well visualised on the previous examination).
  Opinion: No evidence of active perianal sepsis or fistula.”

**Figure 2:**
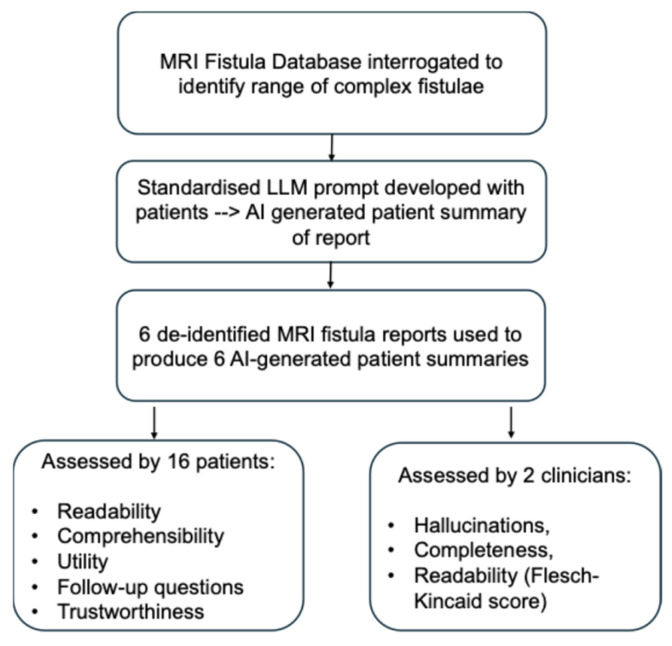
Study Design.

AI-generated patient friendly report:
  “Your MRI scan shows no new changes compared to your previous scan. There is an area in the lower part of the anal canal (from 5 to 7 o’clock) with signs of pastinflammation. This appears to be a chronic issue but not active at this time. The rest of the anal canal and surrounding structures are healthy, with no signs of infection, fluid collections, or fistulas (abnormal passages).
  The rectum shows ongoing inflammation, consistent with a condition called proctitis, but this is stable. There are no signs of infection in deeper areas of the pelvis, and no abnormalities were found in other pelvic organs like the bladder or prostate. Small, simple hernias in the groin area were noted, which were not visible in the last scan.”
  Next Steps:
  No active infection or new problems were found. It is important to continue managing the underlying condition causing the rectal inflammation. Please discuss these findings with your doctor, who may recommend further monitoring or treatments to keep your symptoms under control.”
  Patients rated reports on readability, comprehensibility, utility, quality, follow-up questions, and trustworthiness using 5-point Likert scales (Figure 2). Digital access and perceived usefulness of AI summaries were also assessed. Data distributions were assessed visually; non-parametric Wilcoxon signed-rank tests were used for patient-reported outcomes due to non-normal distributions, while paired t-tests were applied to Flesch-Kincaid readability scores, which approximated a normal distribution. Statistical analysis was performed using IBM SPSS Statistics v29. Ethical approval was granted (REC Reference: 24/WA/0374).

## 3. Results

There were 16 participants in total, with an average age of 40 and an even gender distribution. Among them, 8 patients had perianal fistulizing Crohn’s disease (pfCD), 7 had cryptoglandular fistulae, and 1 had a pouch-related fistula. Participants had undergone an average of 4.5 MRI scans each since being diagnosed with Crohn’s disease.

Regarding digital access, 13/16 (81.3%) reported using the NHS application, while 11/16 (68.8%) found original diagnostic reports somewhat or extremely difficult to understand. Notably, 13/16 patients (81.3%) felt that a patient-friendly summary of their diagnostic report (e.g., MRI scan) would be either extremely useful or very useful.

Patients assessed AI-generated summaries and original reports using a subjective Likert scale from 1 (strongly disagree) to 5 (strongly agree). AI-generated summaries were rated significantly higher for readability, with a median score of 5 (interquartile range [IQR] 3.25–5), compared to a median of 2 (IQR 2–4) for the original reports (*p* = 0.011). Comprehensibility ratings showed a similar pattern, with AI summaries scoring a median of 5 (IQR 3–5) versus 2 (IQR 2–3) for the originals (*p* = 0.007).

Patients rated the ‘overall quality’ (based on patient satisfaction) of reports higher for the AI summaries, with a median score of 4.5, compared to 4 for the original reports (*p* = 0.013), although it is important to note that radiologist reports are intended primarily for clinicians rather than patients. Patients were more likely to have follow-up questions after reading the original report (median score of 4) compared to the AI summary (median score of 3), with a p-value of 0.018, suggesting that AI summaries pre-emptively addressed key concerns. Trustworthiness ratings were similar between both report types, with a median score of 4 for each (*p* = 0.058) (Table 1).

Clinician assessments corroborated these patient findings (Table 2). The AI-generated summaries achieved a significantly higher Flesch-Kincaid reading score (mean 54.6/100) compared to the original reports (32.2/100, *p* = 0.005), indicating improved readability appropriate for a 12+ reading age. Two independent clinicians rated the AI summaries 5/5 for quality based on completeness and clarity of information. Notably, AI-generated summaries were produced rapidly, averaging 18.2 s per report, and no hallucinations or clinically significant inaccuracies were identified.

## 4. Discussion

This pilot study highlights key challenges in patient comprehension of MRI reports and the potential of AI-generated summaries to improve patient communication, even among digitally engaged populations. Despite four-fifths of participants regularly using the NHS application, over two-thirds reported difficulties in understanding diagnostic reports. This reflects a broader problem: diagnostic reports are currently traditionally written by radiologists for clinician-to-clinician communication, using specialized terminology that is opaque to patients. With direct access now standard in many health systems, this disconnect can lead to anxiety, misinterpretation, and disengagement from care [3,4].

Adapting these reports in a practical and meaningful way is crucial to improve the understanding of patients, which has been shown to improve engagement with their treatment and improve overall outcomes. Notably, 81.3% of patients expressed that a patient-friendly summary would be extremely or very useful, reinforcing the need for improved communication tools in pfCD and related conditions.

Our findings show a clear patient appetite for change: over 81% of participants reported they would find a patient-friendly summary extremely or very useful. Quantitative comparisons confirmed that AI-generated summaries substantially outperformed standard reports in readability, comprehensibility, and perceived utility (all *p* < 0.05). The AI summaries also achieved significantly better Flesch-Kincaid readability scores, indicating alignment with an accessible reading level for general audiences. Notably, patients reported fewer follow-up questions after reading AI-generated summaries, suggesting improved clarity and reduced uncertainty.

These findings support existing evidence from other imaging specialties showing that AI-generated, patient-friendly reports can improve understanding and satisfaction [5,6,7]. Importantly, in the context of pfCD, a disease with high emotional burden and frequent need for monitoring, clear communication can have substantial psychological benefits. Patients in our previous PPI survey reported that seeing improvement on MRI brought emotional relief and hope [3]. By making reports more accessible, AI tools can help patients better understand their disease trajectory, potentially improving shared decision-making, treatment adherence, and clinical outcomes [4].

From a clinical perspective, the implications of improved patient-friendly summaries are considerable. Patient-friendly reports would allow patients who would not normally seek out this information to have an option to access it, enhancing patient-clinician communication. For patients who do have access, this may lead to reduced time constraints in clinics and it may empower patients to learn more about their disease. AI-generated summaries may streamline workflow by helping patients with normal MRI findings avoid unnecessary urgent follow-up, while those with concerning findings could be directed to appropriate urgent care pathways more efficiently. Given that AI summaries were generated in just 18.2 s with no hallucinations or inaccuracies, these tools could contribute to faster and more effective patient communication without compromising reliability.

A key strength of this study is its direct patient-centred evaluation. Rather than relying solely on readability algorithms or clinician judgment, we assessed patient perceptions of both original MRI reports and AI-generated summaries in a blinded, structured format. This approach ensures findings are grounded in real-world patient experience, addressing a critical gap in existing research that often emphasises clinician perspectives over patient outcomes.

Additionally, our study leveraged both subjective ratings and objective measures (Flesch-Kincaid scores), providing a robust, multi-dimensional assessment of report quality. The inclusion of quantitative readability analysis demonstrated that AI summaries achieved significantly higher accessibility scores while maintaining accuracy and avoiding hallucinations, a crucial factor for safe deployment.

Despite these promising results, this study has several limitations. The small sample size limits generalizability, and participants were recruited from a single healthcare system with high digital engagement, potentially biasing results toward tech-literate populations. The AI model used had no specific training on IBD-related imaging terminology beyond its pre-existing dataset. Additionally, while no hallucinations were identified in this study, a more comprehensive assessment across a broader dataset is necessary to confirm the reliability of AI-generated content. Traditionally, radiology reports are written for clinicians rather than patients, which may bias findings in favour of AI-generated, patient-friendly summaries. A human-generated patient-friendly summary was not included as a comparator, which may limit contextualization of AI performance; future studies could explore this alongside workflow considerations. This study and future research are likely to highlight essential features that could be integrated into reports, considering that patients may also access them.

To translate these promising findings into clinical practice, larger, adequately powered multi-centre validation studies are needed, as the small sample size may limit statistical power, generalisability, and applicability to patients with lower digital or health literacy. Evidence from allied medical fields suggests that increasing medical complexity and task demands can result in a deterioration in performance [8]. Future research is needed to understand and quantify the rate and severity of hallucinations through larger validation studies incorporating a greater diversity of MRI scans. It will be necessary to explore methods that minimise it, whilst assessing risk both from the patient and healthcare provider point of view, whilst continuously refining AI models based on direct patient feedback (reinforcement learning from human feedback or RLHF). Progress will also depend on models with high stability to avoid catastrophic forgetting in complex, domain-specific tasks [9], alongside tailored biomedical foundation models such as the open-source BiomedGPT, which has shown satisfactory performance with an 8.3% error rate in generating complex radiology reports and may enable task-specific applications with substantially lower error rates [10]. Integration into clinical workflows will also require careful design, ensuring summaries can be appended to existing reports and reviewed without adding burden to radiologists, gastroenterologists, or surgeons. There is also potential for these tools to support patients with limited English proficiency through near-instant translation, though this remains an area for future research. Models should be refined to accommodate individual patient preferences and literacy levels, potentially offering tiered levels of detail. Importantly, any AI-generated content must be validated and contextualised by clinicians to maintain trust and support shared decision-making. Finally, implementation studies should explore the real-world impact of patient-friendly summaries on outpatient workloads, patient engagement, anxiety, treatment adherence, and health outcomes. Future studies should incorporate longitudinal follow-up to assess the impact of AI-generated summaries on patient anxiety, treatment adherence, and clinical outcomes, providing a more comprehensive evaluation of their utility in routine care. Beyond radiology, similar approaches are likely to be explored in other diagnostic specialties such as pathology and endoscopy, where structured reports form a key part of patient care; indeed, work is already underway to investigate this in endoscopy.

## Figures and Tables

**Table 1:** Blinded Patient Assessment of Original MRI report vs. ChatGPT patient-friendly summary (1 = Strongly Disagree, 5 = Strongly agree).

	Original Report	AI-Generated Patient Friendly Summary	*p*-Value *
Readability of report on Likert scale	2 (2–4)	5 (3.25–5)	0.011
Comprehensibility	2 (2–3)	5 (3–5)	0.007
Utility	3 (2–4)	5 (3.25–5)	0.014
Overall quality	4 (2–4)	4.5 (3.25–5)	0.013
Follow up questions	4 (4–5)	3 (2.25–4)	0.018
Trustworthy	4 (3.5–4)	4 (4–5)	0.058

Median scores provided (IQR in brackets). * Wilcoxon-signed rank test.

**Table 2:** Non-patient assessment of Original vs. AI-generated patient-friendly summary.

	Original Report	AI-Generated Patient-Friendly Summary	*p*-Value
Flesch—Kincaid reading score *	32.2/100	54.6/100	0.005
Quality of report	N/A	5/5	
Time to generate	N/A	18.2 s	
Number of Hallucinations	N/A	0	

* A score of 60+ is considered appropriate for a reading age of 12+ years. The paired t-test/Wilcoxon rank non-parametric test was used to compare scores between the 2 groups.

## Data Availability

The original contributions presented in this study are included in the article. Further inquiries can be directed to the corresponding author.

## Appendix A

### Appendix A.1. Example Original Complex Perianal Fistula MRI Report

History of perianal Crohn’s
Updated MRI to rule out collection and to evaluate fistula anatomy

Findings
Comparison is made with previous MR.

There remains an internal opening at 7 o’clock lower rectum that descends and joins and upper canal internal opening that branches to cross the external sphincter at 4 and 6 o’clock, each descending to an external openings at 4 and 7 o’clock near anal verge respectively. The left ischioanal branch now measures up to 15 mm in diameter and the 4 o’clock external opening has narrowed.

No proctitis. Review of the remaining pelvic structures is unremarkable.

No suspicious bone lesions demonstrated.

Impression
Complex high transphincteric fistulas with increase in volume of the left ischioanal fossa tract likely related to partial closure of the draining external opening.

### Appendix A.2. Example AI-Generated Patient-Friendly Summary: Complex Perianal Fistula

Your MRI shows complex fistulas (abnormal passages) related to Crohn’s disease. There are two internal openings: one at the 7 o’clock position in the lower rectum and one higher in the anal canal. These connect to tracts that cross the external sphincter at the 4 and 6 o’clock positions and exit at the skin near the anal verge at 4 and 7 o’clock, respectively.

The left ischioanal branch (part of the fistula system) has increased in size to 15 mm, likely due to partial closure of the external opening at 4 o’clock, which is now narrower. There are no signs of inflammation in the rectum (no proctitis) or any issues with other pelvic structures.

Next Steps:

This MRI shows a complex fistula system with some changes in size. Please discuss these findings with your doctor, as further treatment may be needed to manage the fistulas and address the partially narrowed opening. Options could include drainage, seton placement, or other therapies to prevent further complications. Regular follow-up will also be important.
